# Terapia Herbal na Pós-Menopausa

**DOI:** 10.36660/abc.20250502

**Published:** 2025-10-20

**Authors:** Rosana dos Santos e Silva Martin, Luis Cuadrado Martin

**Affiliations:** 1 Universidade Estadual Paulista Júlio de Mesquita Filho Faculdade de Medicina Botucatu SP Brasil Universidade Estadual Paulista Júlio de Mesquita Filho Faculdade de Medicina – Câmpus de Botucatu, Botucatu, SP – Brasil; 2 Universidade Estadual Paulista Botucatu SP Brasil Universidade Estadual Paulista (UNESP), Botucatu, SP – Brasil

**Keywords:** Menopausa, Hipertensão Arterial, Medicina Tradicional Chinesa

A medicina tradicional chinesa e seus produtos modernos demonstraram potencial no tratamento da aterosclerose. Os possíveis mecanismos incluem a regulação dos lipídios sanguíneos, antiperoxidação lipídica, antipolimerização, anticoagulação e pró-fibrinólise, inibição da proliferação de células musculares lisas e proteção da função endotelial vascular.^1^ Derivados do ginseng estão sendo utilizados no tratamento da menopausa^2^ e da andropausa.^3^

O artigo "Tratamento Crônico com Panax ginseng e Angelica keiskei Reduz a Pressão Arterial e Melhora a Função Endotelial em Ratas Ovariectomizadas", publicado neste periódico,^4^ obteve melhora na pressão arterial em ratas ovariectomizadas tratadas com Panax ginseng e Angelica keiskei. Este efeito foi associado à melhora da função endotelial.^4^

Ginsenosídeos (de Panax ginseng),^2^ assim como chalconas, cumarinas e flavononas (de Angelica keiskei), são substâncias com propriedades documentadas de redução de inflamação, estresse oxidativo, glicose, fibrose e pressão arterial, além de propriedades antimicrobianas, antitumorais, laxativas e galactagogas.^5 , 6^

Panax ginseng é uma planta antiga com usos medicinais enraizados na Medicina Tradicional Chinesa. Esta planta ativa a enzima eNOS (óxido nítrico sintase endotelial), que converte L-arginina em L-citrulina e NO, levando à ativação da enzima guanilato ciclase solúvel no músculo liso, induzindo vasorrelaxamento e, consequentemente, diminuição da pressão arterial.^2^

Os flavonoides presentes na Angelica keiskei possuem uma estrutura complexa que inibe a enzima conversora de angiotensina, uma enzima essencial na regulação da pressão arterial. Estudos com extratos de Angelica keiskei indicam que eles exercem efeitos protetores vasculares contra a vasoconstrição induzida pela fenilefrina por meio de mecanismos como o NO e o fator relaxante derivado do endotélio.^7 , 8^

O estudo em questão não foi capaz de diferenciar os efeitos de cada componente da associação de ervas. Além disso, não é impossível que o efeito observado seja atribuível a apenas uma das substâncias testadas, visto que Panax ginseng e Angelica keiskei foram utilizados em associação, e nenhum estudo adicional foi realizado com cada fitoterapia isoladamente.

A inflamação e o estresse oxidativo estão envolvidos em múltiplos processos fisiopatológicos,^2^ culminando na disfunção endotelial. Por outro lado, distúrbios da função endotelial estão envolvidos na patogênese da hipertensão.^3 , 4^ O declínio hormonal feminino é apontado como causa de disfunção endotelial e hipertensão em mulheres na pós-menopausa. Além disso, a hipertensão que acompanha a menopausa pode causar diversas complicações cardiovasculares. Portanto, se espera que a suplementação hormonal possa mitigar o alto risco cardiovascular do período pós-menopausa.

No entanto, muitos ensaios clínicos experimentais não conseguiram demonstrar o efeito da terapia hormonal na atenuação do risco cardiovascular em mulheres na pós-menopausa.^9 , 10^ Esses ensaios utilizaram hormônios animais ou análogos sintéticos para reposição. Existem vários análogos vegetais para hormônios femininos. Esses análogos não foram testados neste contexto clínico de pacientes. Outras terapias fitoterápicas têm o potencial de reverter o aumento desvantajoso da inflamação, do estresse oxidativo e da disfunção endotelial.

Os resultados do artigo apresentado têm implicações fisiopatológicas e abrem a possibilidade da terapia fitoterápica para mitigar e prevenir doenças cardiovasculares em mulheres na pós-menopausa. Portanto, esta linha de pesquisa tem o potencial de contribuir para a compreensão do tratamento de humanos com essa entidade nosológica. Vale ressaltar que já existem evidências para a profilaxia da hipertensão e o uso dessa terapia fitoterápica em humanos.

É importante ressaltar que a terapia com estrogênio tem o mesmo efeito sobre a disfunção endotelial e a pressão arterial que a fitoterapia, mas, quando testada em ensaios clínicos com mulheres, o desfecho cardiovascular não melhorou nem piorou. Assim como a terapia com estrogênio, a fitoterapia para mulheres na menopausa precisa ser testada em ensaios clínicos com humanos. O presente artigo incentiva ensaios clínicos, mas não indica qualquer uso clínico desta fitoterapia.

## References

[1] Nijat D, Zhao Q, Abdurixit G, He J, Liu H, Li J (2025). An Up-to-Date Review of Traditional Chinese Medicine in the Treatment of Atherosclerosis: Components, Mechanisms, and Therapeutic Potentials. Phytother Res.

[2] Fan S, Zhang Z, Su H, Xu P, Qi H, Zhao D (2020;). Panax Ginseng Clinical Trials: Current Status and Future Perspectives. Biomed Pharmacother.

[3] Park SY, Lee YS, Heo I, Myung SC, Lee SJ, Kim JW (2025). Efficacy and Safety of Ginseng Berry Extract (SIRTBERRY™) in Treating Andropause Symptoms: A Randomized, Double-Blind, Placebo-Controlled Clinical Trial. World J Mens Health.

[4] Silva NF, Moraes LHO, Sabadini CP, Alcântara RCC, Dias PC, Rodrigues GJ (2025). Chronic Treatment with Panax ginseng and Angelica keiskei Decreases Blood Pressure and Improves Endothelial Function in Ovariectomized Rats. Arq Bras Cardiol.

[5] Yang J, Jiang W, Park JH, Seong ES, Kwon YS, Kim MJ (2025). Antioxidant and Pancreatic Lipase Inhibitory Activities of Panax Japonicus (T. Nees) C.A. Meyer. Plants.

[6] Karimi M, Barjasteh AH, Shariatzadeh M, Taha SR, Fazlollahpour-Naghibi A, Rezaei P (2025;). Ginsenosides and Gastrointestinal Cancers: A Novel Therapeutic Strategy in Cancer Therapy. Pathol Res Pract.

[7] Wahyuni I, Aulifa DL, Rosdianto AM, Levita J (2024). The Pharmacology Activities of Angelica Keiskei Koidzumi and its Efficacy and Safety in Humans. Heliyon.

[8] Ohta M, Fujinami A, Oishi K, Kobayashi N, Ohnishi K, Ohkura N (2019). Ashitaba (Angelica Keiskei) Exudate Prevents Increases in Plasminogen Activator Inhibitor-1 Induced by Obesity in Tsumura Suzuki Obese Diabetic Mice. J Diet Suppl.

[9] Garza NYC, Rivas JAD, Arce CF, Avila MEF, Castro CS, González SAV (2025). Cardiovascular Outcomes of Menopause Hormone Therapy Initiated in Women Aged ≥60 Years or ≥10 Years Post-Menopause: A Systematic Review of the Literature. Post Reprod Health.

[10] Johansson T, Karlsson T, Bliuc D, Schmitz D, Ek WE, Skalkidou A (2024). Contemporary Menopausal Hormone Therapy and Risk of Cardiovascular Disease: Swedish Nationwide Register Based Emulated Target Trial. BMJ.

