# Supplementary material for: Terapia Herbal na Pós-Menopausa
Source: Arq Bras Cardiol. 2025 Oct 20;122(8):e20250502. [Article in Portuguese] doi: 10.36660/abc.20250502 (PMC12671723; doi:10.36660/abc.20250502)
Supplement: Translation

# Translation: Herbal Therapy in Post-Menopause

**DOI:** 10.36660/abc.20250502i

**Authors:** Rosana dos Santos e Silva Martin, Luis Cuadrado Martin

**Affiliations:** 1 Botucatu SP Brazil Universidade Estadual Paulista Júlio de Mesquita Filho Faculdade de Medicina – Câmpus de Botucatu, Botucatu, SP – Brazil; 2 Botucatu SP Brazil Universidade Estadual Paulista (UNESP), Botucatu, SP – Brazil

**Keywords:** Menopause, High Blood Pressure, Traditional Chinese Medicine

Traditional Chinese medicine and its modern products have shown potential in treating atherosclerosis. The possible mechanisms include regulation of blood lipids, anti-lipid peroxidation, anti-polymerization, anticoagulation, and pro-fibrinolysis, inhibition of smooth muscle cell proliferation, and protection of vascular endothelial function.^1^Ginseng derivatives are being used in menopause^2^treatment and andropause treatment.^3^

The paper "Chronic Treatment with Panax ginseng and Angelica keiskei Decreases Blood Pressure and Improves Endothelial Function in Ovariectomized Rats", published in this journal,^4^obtained improvement in blood pressure in ovariectomized rats treated by Panax ginseng and Angelica keiskei This effect was associated with amelioration of endothelial function.^4^

Ginsenosides (from Panax ginseng),^2^as well as chalcones, coumarins, and flavanones (from Angelica keiskei), are substances with documented properties of reduction of inflammation, oxidative stress, glycosis, fibrosis, and blood pressure, in parallel with antimicrobial, antitumor, laxative, and galactagogue properties.^5 , 6^

Panax ginseng is an ancient plant with medicinal uses rooted in Traditional Chinese Medicine. This plant activates the enzyme eNOS (endothelial nitric oxide synthase), which converts L-arginine into L-citrulline and NO, leading to activation of the soluble guanylate cyclase enzyme in smooth muscle, inducing vasorelaxation and, consequently, a decrease in blood pressure.^2^

Flavonoids in Angelica keiskei possess a complex structure that inhibits angiotensin-converting enzyme, a key enzyme in regulating blood pressure. Angelica keiskei extracts, studies indicate that they exert vascular protective effects against phenylephrine-induced vasoconstriction through NO and endothelium-derived relaxing factor mechanisms.^7 , 8^

The study in question was not capable of differentiating the effects of each component of the herbal association. Furthermore, it is not impossible that the observed effect would be attributable to only one of the tested substances, as Panax ginseng and Angelica keiskei were used in association, and no additional study was performed with each herbal therapy isolated.

Inflammation and oxidative stress are involved in multiple pathophysiological processes,^2^culminating in endothelial dysfunction. On the other hand, disorders of endothelial function are involved in the pathogenesis of hypertension.^3 , 4^Female hormonal decline is implicated as the cause of endothelial dysfunction and hypertension in post-menopausal women. Moreover, the hypertension that accompanies menopause can cause many cardiovascular complications. Therefore, it would be expected that hormonal supplementation should mitigate the high cardiovascular risk of the post-menopausal period.

Nevertheless, many investigational trials failed to demonstrate the effect of hormonal therapy to attenuate cardiovascular risk in post-menopausal women.^9 , 10^These trials used animal hormones or synthetic analogues for replacement. There are several vegetable analogs to female hormones. These analogues were not tested in this clinical setting of patients. Other herbal therapies have the potential to reverse the disadvantageous increase in inflammation, oxidative stress, and endothelial dysfunction.

The results of the presented paper have pathophysiological implications and open the possibility of herbal therapy to mitigate and prevent cardiovascular disease in post-menopausal women. Therefore, this line of research has the potential to bring contributions to understanding the treatment of humans with this nosological entity. It is of note that already there is evidence for the prophylaxis of hypertension, the use of this herbal therapy in humans.

It is of note that estrogen therapy has the same effect on endothelial dysfunction and blood pressure as herbal therapy, but when tested in clinical trials in women, the cardiovascular outcome did not improve or worsen. Like estrogen therapy, herbal therapy for menopausal women needs to be tested in human clinical trials. The present paper encourages clinical trials, but does not indicate any clinical use of this herbal therapy.
